# Experimental Investigation on the Mechanical Properties and Microstructure of Basalt Fiber Reinforced Engineered Cementitious Composite

**DOI:** 10.3390/ma13173796

**Published:** 2020-08-28

**Authors:** Qiang Du, Changlu Cai, Jing Lv, Jiao Wu, Ting Pan, Jie Zhou

**Affiliations:** 1 School of Economics and Management, Chang’ an University, Xi’an 710064, China; q.du@chd.edu.cn (Q.D.); jiao_wu@chd.edu.cn (J.W.); 2 Center for Green Engineering and Sustainable Development, Chang’ an University, Xi’an 710064, China; 3 School of Civil Engineering, Chang’ an University, Xi’an 710064, China; lvjing21@chd.edu.cn (J.L.); ting_pan@chd.edu.cn (T.P.); jie_zhou@chd.edu.cn (J.Z.)

**Keywords:** BF-ECC, water-binder ratio, fly ash, mechanical properties, microstructure

## Abstract

This study investigated fundamental mechanical properties of a basalt fiber reinforced engineered cementitious composite (BF-ECC) with different volume fractions of basalt fiber (BF), water–binder ratio (W/B) and fly ash (FA) content. The compressive strength, splitting tensile strength, flexural strength and static modulus of BF-ECC were studied at 3, 28 and 56 days, respectively, to explore their development along the ages. Furthermore, the scanning electron microscopy (SEM) analysis was conducted to evaluate the microstructure of BF-ECC. Experiment results demonstrated that bond quality between the BF and the matrix is good, which leads to a significant increase in the flexural strength and splitting tensile strength. The pozzolanic effect of FA obviously improved the splitting tensile and flexural strength of BF-ECC after 56 days of curing, and the appropriate content of the FA content in the BF-ECC ranges from 50% to 60%.

## 1. Introduction

Engineered cementitious composite (ECC) is a cement-based material consisting of cement, fly ash (FA), sand, water, a chemical admixture and short discrete fibers [1]. ECC is known for its enhanced tensile ductility, with the tension strain ranging from 3% to 8% and the width of multi-cracks usually being less than 200 μm [2,3,4]. Due to these special advantages, ECC is widely used, especially in structures that require an enhanced tensile capacity, such as retaining walls, dams, aqueducts, bridge decks and building dampers [5].

Various types of fibers have been used in ECC, such as polyethylene (PE), polypropylene (PP) and polyvinyl alcohol (PVA) fibers. These fibers have been shown to effectively improve the mechanical properties of the ECC [6,7,8]. Nevertheless, among all the currently used fibers, PE fibers are expensive and do not adhere sufficiently to the cementitious matrix. PP fibers show some disadvantages in the tensile strength and Young’s modulus. Although PVA fibers are widely used in ECC due to some special advantages, such as good hydrophilic nature and higher tensile strength [9,10,11], the fibers still significantly increase the material cost of an ECC. Above all, PP, PE and PVA fibers are all organic polymer fibers, so they have the common disadvantage of poor high temperature resistance. For example, some studies showed that the operating temperature of a PVA-ECC can only reach 200 °C [12,13], which poses a great safety risk when it is applied.

Basalt fibers (BF) are a new type of inorganic fiber produced by melting basalt at high temperatures (approximately 1200 °C to 1500 °C) [14,15]. They have excellent resistance to high temperatures [15]. Below 200 °C, the average tensile strength of BF is hardly affected [16]. Above 200 °C, the strength reduction of BF is less significant when compared with that of carbon fibers and glass fibers. In addition, BF can still maintain an appreciable volumetric integrity at high temperatures (600–1200 °C) [17]. Furthermore, they are relatively inexpensive and ecologically safe due to their manufacturing process, which does not require a substantial amount of energy and chemical additives to obtain an appropriate thermal strength and crystal structure [18]. Owing to the contents of SiO_2_, Al_2_O_3_, MgO, CaO and TiO_2_ in basalt, BF have good chemical stability, chemical resistance, corrosion resistance and water resistance [15,19]. Other advantages, such as a high Young’s modulus, uniform dispersion and good compatibility with cement-based materials, have also been widely recognized [14,20]. These advantages enable BF to attract increasing attention as competitive and suitable additions to composite materials [14,15].

The influence of BF on concrete was revealed by Wang et al. [21], the results showed that the compressive strength, splitting tensile strength and flexural strength are effectively enhanced with the increase of BF (0.1%, 0.15% and 0.2%). Similar evidence was provided by Jiang et al. [22], and the corresponding results indicated that the addition of BF with a volume fraction from 0.05% to 0.3% produced a higher increase than PP fibers in terms of both the tensile and flexural strengths. The dynamic behavior of a BF reinforced natural hydraulic mortar was also studied. The results indicated that adding fibers with a volume fraction of 1% or 2% led to an increased toughness and ductile behavior of the mortar [23]. Jiang et al. [24] similarly demonstrated that the resistance to shock, toughness and plastic shrinkage cracking of the mortar can be increased by the addition of BF (0%, 0.03%, 0.06%). However, the research studies focused on mechanical properties of ECC reinforced with BF are not adequate. To facilitate their use in ECC, it is essential to comprehensively investigate the performance of basalt fiber reinforced engineered cementitious composite (BF-ECC).

For optimizing the mechanical properties of ECC, the compressive strength, splitting tensile strength, flexural strength and static modulus were tested in three perspectives: fiber volume fraction (0–2%), water–binder ratio (0.28–0.40) and FA content (30–70%). At the same time, the bonding performance, fiber dispersion and fiber failure mode were further demonstrated by microscopic analysis.

## 2. Experimental Program

### 2.1. Materials

In this paper, the input materials used to prepare ECC were Ordinary Portland cement, fine sand, FA, water, a polycarboxylic-ether type high-range water reducing admixture (HRWR) and BF. Fine sand employed in this research has a maximum grain size of 118 μm and a fineness modulus of 1.79. As a supplementary cementitious material, class F (grade-I) FA was used to improve the late-age strength of the specimens. HRWR was applied to improve the workability and rheological properties of mixture for an improved fiber distribution. Table 1 provides a summary of the chemical and physical properties of the cement and FA obtained by experiments. The detailed properties of the BF provided by the manufacturer are listed in Table 2.

### 2.2. Mixture Proportions

In this experiment, the fiber volume fraction, W/B and FA content were selected as variable parameters, and three ages of 3, 28 and 56 days were set, so as to explore the compressive strength, splitting tensile strength, flexural strength and static modulus of basalt fiber reinforced engineered cementitious composite (BF-ECC). In this way, three groups of mixtures were prepared. Each group of experiments is repeated three times to ensure the reliability of the data obtained. Therefore, 36 specimens were prepared for each mix proportion. In the first set, the effect of different volume fractions of the BF (0%, 0.5%, 1%, 1.5% and 2%) on the BF-ECC was investigated for constant W/B (0.28) and FA contents (60%). In the second set, the influence of different W/B (0.28, 0.32, 0.36 and 0.40) was carried out for a constant BF volume fraction (2%) and FA content (60%). The third set of experiments explored the effect of different FA contents (30%, 40%, 50%, 60% and 70%) on the BF-ECC under the condition that the BF volume fraction and W/B were constant at 2% and 0.28, respectively. The details of the mix proportion of BF-ECC designed for this paper are shown in Table 3. The materials and mix design proportion for the BF-ECC was selected based on the standard design of ECC_M45 [1].

### 2.3. Mixing Procedure and Sampling

Cubes with dimensions of 100 mm × 100 mm × 100 mm were designed as the specimens for the compressive strength test. The splitting tensile strength was tested with 200 mm height and 100 mm diameter cylinders. Prism specimens (100 mm × 100 mm × 400 mm) were prepared for the flexural strength test. For the static modulus test, specimens with cylinders that have a 300 mm height and 150 mm diameter were employed.

The mixing of the specimens is carried out in a standard mixer. First, cement, FA and sand were blended for approximately 2 min. Then, the BF were added manually and gradually, and the mixture was mixed for another 3 min to ensure a reasonable dispersion. After that, water and HRWR were slowly added, and adequately mixed all input materials for 3–6 min. Finally, the mixtures were formed in a mold and demolded after 24 h. All specimens were placed in a curing chamber with 95 ± 5% relative humidity (RH) and a temperature of 20 ± 2 °C until the predetermined testing ages were reached.

### 2.4. Sample Testing Method

The compressive strength test was performed using a 300 kN hydraulic compression testing machine (Wuxi Jianyi, Jiangsu, China) with a rate of 3 kN/s. BS EN 12390-3:2009 standard [25] was used as the basis for this test. The splitting tensile test was conducted on the same machine with a rate of 0.4 kN/s. Using an electronic universal testing machine (Wuxi Jianyi), the flexural strength test was performed under displacement control with a rate of 2 kN/s. This experimental setup was recommended in the BS EN 12390-5: 2009 standard [26]. The static modulus was determined according to BS EN 1881-121: 1983 [27] with a rate of 1 kN/s. Two dial gauges were fixed on both sides of cylinders to record the corresponding movements during testing. According to the GB/T 20307-2006 standard [28], the microstructure of BF-ECC was evaluated by the S4800 field-emission scanning electron microscope (Hitachi, Tokyo, Japan).

## 3. Results and Discussion

### 3.1. Effect of the Volume Fraction of BF on the Mechanical Properties

**Figure 1:**
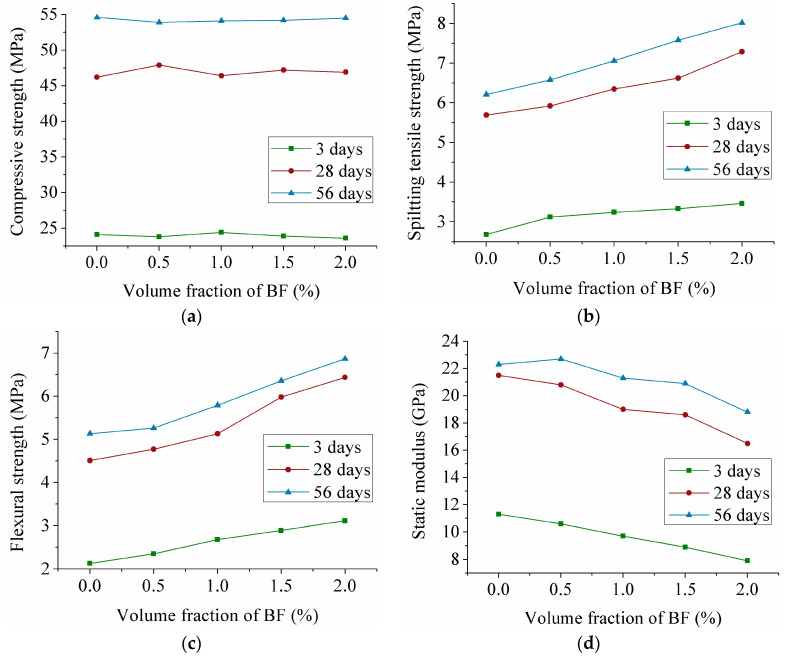
Mechanical properties of ECC for different volume fraction of BF. (**a**) Effect of the volume fraction of BF on the compressive strength; (**b**) Effect of the volume fraction of BF on the splitting tensile strength; (**c**) Effect of the volume fraction of BF on the flexural strength; (**d**) Effect of the volume fraction of BF on the static modulus.

The relationship between the volume fraction of BF and the mechanical properties of ECC at 3, 28 and 56 days is displayed in Figure 1. As expected, the change of compressive strength is not obvious with an increase in fiber volume fraction in Figure 1a. For the PVA-ECC, the combined effects of the fibers resulted in the decrease of the compressive strength as fiber volume fraction increased [29]. The negative effect of PE fibers on compressive strength of ECC is obvious, an increase in fiber volume fraction from 1% to 1.5%, 2% and 2.5% showed a loss in compressive strength of about 8%, 19%, 28%, and 33%, respectively [30]. Obviously, adding BF would not be an effective method for increasing the compressive strength. However, compared with that for PVA and PE fibers, BF should not cause a significant decline in the compressive performance.

Figure 1b shows that with increasing the fiber volume fraction, the splitting tensile strength rises at all ages. This is consistent with the research results presented by Said and Razak [30]. As BF volume fraction changes from 0% to 2%, the splitting tensile strength at 28 and 56 days increases 28% and 29%, respectively. The splitting tensile strength changes for ECC at 3 days is found to be different from that at 28 and 56 days as it has a flatter ascending trend. The splitting tensile strength of ECC with 2% fiber volume fraction is also 29% higher than that of the nonfiber specimens at 3 days. However, compared with BF, the increase of PVA fiber volume fraction made ECC show a greater increase in tensile strength [31]. The strength reduction coefficient of PVA fibers is lower than that of BF and maybe that is the cause.

The conclusion can be drawn from Figure 1c that the flexural strength also improves as the BF volume fraction increases from 0% to 2%. At 3, 28 and 56 days, compared to that of the 0% fiber specimens, the flexural strength of ECC with 2% BF increases 46%, 43% and 34%, respectively. Furthermore, at 3, 28 and 56 days, the flexural strength of ECC with 2% BF increases by 32%, 35% and 31%, respectively, compared with that for the 0.5% fiber specimens. Atahan et al. [32] claimed that the flexural strength of PVA-ECC improved by 20% at 2% fiber volume fraction compared to that of PVA-ECC with 0.5% fiber volume fraction. Furthermore, when PVA fiber volume fraction changed from 0% to 1.2% and 1.4%, the flexural strength first decreased to 5.18MPa, and then rose slightly [29]. It is obvious that BF are more effective than PVA fibers in improving the flexural strength. The reason may be that the Young’s modulus of BF is much higher than that of PVA fibers. Fibers with a larger Young’s modulus have a higher peak stress for degumming, which was confirmed by Sasmal et al. [33].

Figure 1a–c show that the BF are particularly effective in enhancing the splitting tensile and flexural strength, but not as effective in enhancing the compressive strength. The reason may be that the influence of fibers on strength is not unidirectional. The bridging effect of fibers can be improved by increasing the fiber volume fraction. The fibers in the matrix are randomly distributed in three dimensions, and thus micro cracks in the early stage are suppressed effectively by the bridging of the BF. When the specimens are cracked, the stress is passed on to the fibers around the micro cracks by bridging. On the contrary, the addition of fiber will increase the interface of the matrix, and led to some voids in interfacial transition zone, resulting in a decrease of matrix density. For the splitting tensile and flexural strength, the positive effect of fibers is dominant, making they rise with the increase of fiber content. However, the positive effect on compressive strength is limited, because the fiber works in tension. Although BF provides a positive impact on compressive strength, the compactness of ECC has also decreased due to an increase in the number of voids when the BF is present [34].

The static modulus of ECC with different volume fractions of BF is shown in Figure 1d. The static modulus trend for the specimens at 28 and 56 days is almost the same. However, it is observed that the static modulus at 3 days shows a linear dependence on the volume fraction of BF, showing a different trend from those at 28 and 56 days. As the volume fraction of BF increases from 0% to 2%, the static modulus at 3, 28 and 56 days decreases by 30%, 23% and 16%, respectively. The reason may be that the incorporation of fibers inevitably increases the number of voids inside the matrix, resulting in an increase in the porosity of the matrix.

### 3.2. Effect of the W/B on the Mechanical Properties

**Figure 2:**
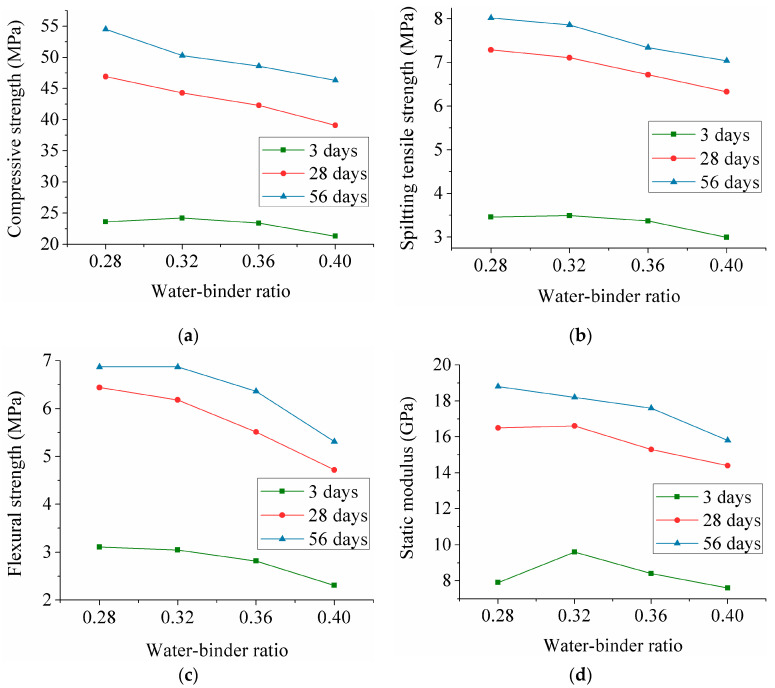
Mechanical properties of basalt fiber reinforced engineered cementitious composite (BF-ECC) for different W/B. (**a**) Effect of the W/B on the compressive strength; (**b**) Effect of the W/B on the splitting tensile strength; (**c**) Effect of the W/B on the flexural strength; (**d**) Effect of the W/B on the static modulus.

The changes in the mechanical properties of the mixtures having different W/B are shown in Figure 2. As expected, the increase of the W/B negatively influenced the compressive strength. In Figure 2a, at 3, 28 and 56 days, the compressive strength of the cube specimens at W/B of 0.40 decreases 10%, 17% and 15%, respectively, over that of BF-ECC with a W/B of 0.28. The same trend exists for the compressive strength of BF-ECC at 28 and 56 days. It can be seen from past studies that W/B has a stronger impact on the compressive strength of PVA-ECC than BF-ECC at 28 days. As W/B changes from 0.33 to 0.38, the compressive strength of PVA-ECC increases by 8% [35]. In this paper, When W/B increases from 0.32 to 0.40, the same result can be obtained.

As shown in Figure 2b, the influence of the W/B on the splitting tensile strength is found to be similar to that for the compressive strength. When the W/B changes from 0.28 to 0.40, the splitting tensile strength decreases by 14%, 13% and 12% at 3, 28 and 56 days. The impact of different W/B on the flexural strength is displayed in Figure 2c, which indicates that the flexural strength is reduced with increasing W/B. Compared with the specimens having W/B of 0.28, increasing the W/B to 0.40 decreases the flexural strength by 26%, 27% and 23% at 3, 28 and 56 days, respectively. More significantly, when the W/B increases from 0.28 to 0.32, the flexural strength of PVA-ECC at 28 days decreases by 12% [29], while BF-ECC only decreases by 4%.

In conclusion, the increase of W/B significantly decreases the strength of BF-ECC. This phenomenon is mainly caused by two reasons. On the one hand, the W/B increment contributes to increased free water, and then a certain number of microscopic pores are produced in the matrix, which causes a decrease in the BF-ECC strength. On the other hand, the increase of W/B decreases bonding performance between the fibers and the matrix. Therefore, the stress that the matrix passes to the fibers is lowered, resulting in a decrease in the strength. Under the combined effect of these two reasons, the strength decreases with increasing W/B.

Figure 2d displays the influence of the W/B on the static modulus. The static modulus also similarly decreases at higher W/B. The 3 days static modulus of BF-ECC with a W/B of 0.28, 0.32, 0.36 and 0.40 varies between 7.6 GPa and 9.6 GPa. At 28 and 56 days, compared to the specimens with a W/B of 0.28, the static modulus of BF-ECC with a W/B of 0.40 decreases by approximately 13% and 16%, respectively. Furthermore, the static modulus trend at 3 and 28 days is different to that at 56 days. When W/B changes from 0.28 to 0.40, the static modulus at 3 and 28 days first increases to 9.6 GPa and 16.6 GPa, respectively, and then shows a sustained drop. This may be due to the hydration of FA.

### 3.3. Effect of the FA Content on the Mechanical Properties

**Figure 3:**
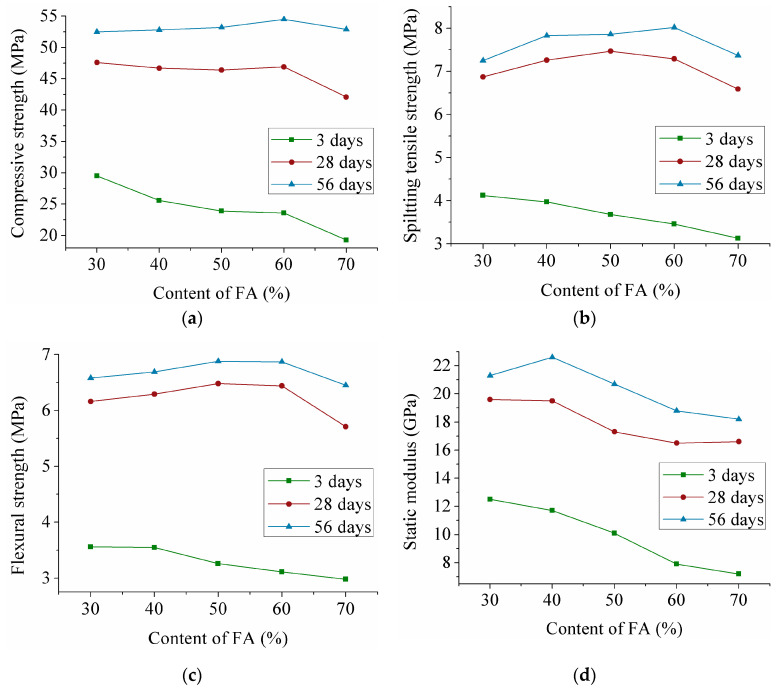
Mechanical properties of BF-ECC for different FA content. (**a**) Effect of the FA content on the compressive strength; (**b**) Effect of the FA content on the splitting tensile strength; (**c**) Effect of the FA content on the flexural strength; (**d**) Effect of the FA content on the static modulus.

Figure 3 shows the effect of the change in FA content on the compressive strength, splitting tensile strength, flexural strength and static modulus of BF-ECC. As presented in Figure 3a, at 28 and 56 days, the variation in the FA content from 30% to 60% does not contribute to a marked improvement in the compressive strength. However, the 3 days compressive strength clearly decreases when the FA content increases, which is due to the reduced level of early age hydration in the Portland cement under the influence of the FA. Furthermore, the compressive strength at all ages decreases obviously when the FA content increases from 60% to 70%. Significantly, the compressive strength at 3 and 28 days decreases by 4.3 MPa and 4.8 MPa, respectively. This result can also be observed in other studies; Sahmaran et al. [36] reported that as FA content increased from 55% to 80%, the compressive strength at 28 days showed continued decline.

At 28 days, related research found that the FA content in PVA-ECC increased by 14% (55% to 69%), resulting in a slight decrease in tensile strength (within 15%), which is consistent with the results of this paper [36,37]. Based on the curves in Figure 3b, the values of 28 days splitting tensile strength range from 6.59 to 7.47 MPa and those at 56 days vary from approximately 7.25 to 8.02 MPa. With an increase in the FA content, the splitting tensile strength increases first and then drops at 28 and 56 days. However, at 3 days, increasing the FA content has a negative impact on the splitting tensile strength, as BF-ECC with a 70% FA content has a 24% drop in the splitting tensile strength compared to the specimens with a 30% FA content.

The effect of the FA content on the flexural strength of BF-ECC is shown in Figure 3c. For specimens at 3 days, a negative correlation is found between FA content and the flexural strength. However, the trend of flexural strength is consistent at 28 and 56 days. When the FA content increases from 30% to 70%, the flexural strength rises slightly and then drops to a small extent. As it increases to 70%, the flexural strength decreases by 12% compared to the specimen with 50% FA at 28 days.

FA is considered to be a beneficial component of ECC, because it can effectively increase the later strength through the pozzolanic effect. Although the slow reaction of the pozzolanic effect is not conducive to the development of an early strength in cement paste, resulting in the early strength decreasing with increasing FA content, its positive effect on the later strength of ECC is more significant. The pozzolanic effect of FA can be seen from Figure 3a–c. It is clear that 3 days strength continues to decrease as the FA content increases, which is different from the strength trend at 28 and 56 days. The main cause of this phenomenon is that the active components in FA gradually react with Ca(OH)_2_ with the increase of age, which effectively improves the growth rate of the matrix strength and endows the BF-ECC with a good strength in the late stages. However, excessive cement replaced by FA causes an insufficient hydration reaction, leading to a decrease in the strength. From the strength curves, the appropriate FA content of BF-ECC is 50% to 60%.

At 3 days, Figure 3d shows that the static modulus steadily decreases as the FA content increases from 30% to 70%. The 28 days curve of the static modulus gradually drops to 16.5 GPa and then rises slightly. The trend of the static modulus at 56 days is different from that at 3 or 28 days. Along with an increase in the FA content, a static modulus peak appears when the FA content is 40%. By comparing the static modulus of the specimen mixed with 40% FA, the result shows that the static modulus of BF-ECC with an FA content of 70% decreases by 19%.

### 3.4. Effect of the Age on the Mechanical Properties

Figure 1, Figure 2 and Figure 3 show that the mechanical properties growth at an early age is higher than that at a late age. The compressive strength, splitting tensile strength, flexural strength and static modulus increase nearly 2 times with age from 3 days to 28 days. However, from 28 days to 56 days, the mechanical properties change slightly. Perhaps the main reason centers on the interfacial aging between fibers and the matrix, which causes a drop in the bonding behavior of the substrate.

### 3.5. Morphology

**Figure 4:**
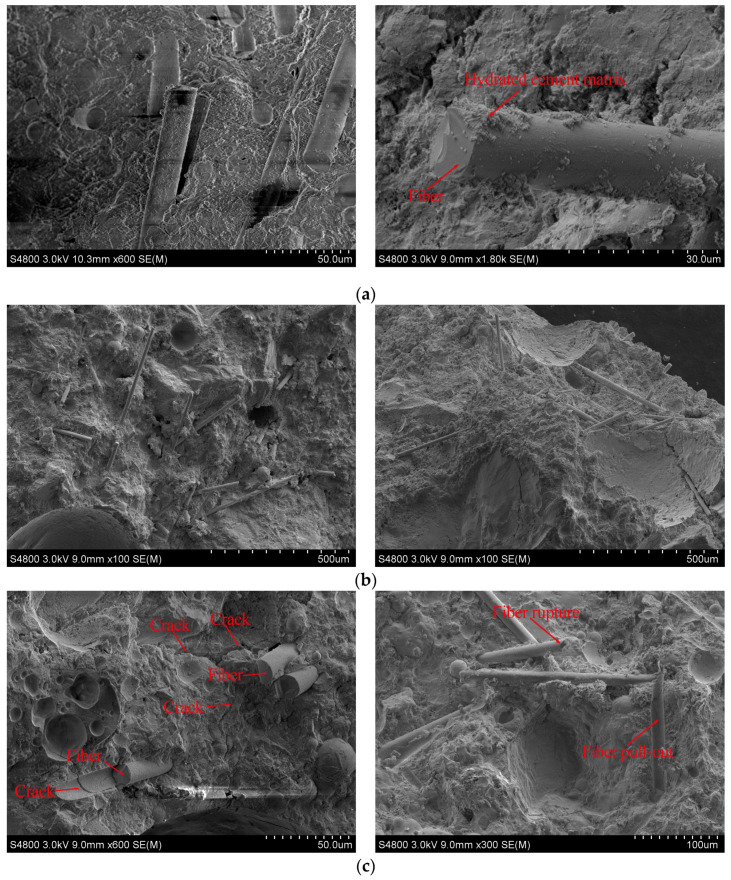
Scanning electron microscope images of BF-ECC. (**a**) SEM photomicrographs of BF reinforced cement paste; (**b**) SEM photomicrographs of BF dispersion; (**c**) SEM photomicrographs of BF damage.

SEM is regarded as a useful technique to understand the microstructural morphology of ECC. To recognize the bonding behavior of the BF and cement matrix, the microstructure of ECC with a 1% volume fraction of BF was observed with SEM. Figure 4a shows the micrographs of the microstructure of the fiber surface and hydrated cement matrix. The dense hydrated cement matrix is attached to the fibers surface. It implies that bonding performance of the fiber and matrix is as expected and desired, while it has been found that better compact fiber/matrix interfacial zone can be formed between BF and matrix than PVA fibers [38]. The hydrophilic surface of BF is the cause of this, therefore, cement paste near the BF has better hydration than that of PVA fibers, and the space between BF and matrix is filled better. Based on Figure 4b, it can be seen that the fibers show the uniform distribution in the matrix during mixing, and they are evenly oriented. The presence of microstructure imperfections in Figure 4b, including matrix flaws, is possible sources of the irregular variations that were observed in the mechanical properties. Furthermore, it can be clearly observed in Figure 4c that the failure mode of the fibers on the fracture surface of BF-ECC is mainly pull-out and rupture. Most fracture surfaces of the fibers that are pulled out from the matrix are neat or slightly frayed, with tiny matrix particles adhering to the smooth fiber surface. The failure of the fiber is mainly caused by extreme energy consumption (during fibers pull-out) and excessive shear friction (during fibers rupture) [39]. The characteristics of fiber bridging are responsible for improving the ECC strength [40,41,42]. Due to the fiber bridging, crack propagation is controlled by fibers, and this enhancement mechanism can be easily observed in Figure 4c.

## 4. Conclusions

This study examined fundamental mechanical properties of BF-ECC through a series of experiments and SEM. The influences of fiber volume fraction, W/B, and FA content on the compressive strength, splitting tensile strength, flexural strength and static modulus were determined and discussed. According to the above investigation, the following conclusions can be drawn:

(1) Incorporating BF is not an effective approach to enhance compressive strength of ECC mixtures. Adding 2% BF in ECC leads to an increase of more than 28% and 34%, respectively, on the splitting tensile strength and flexural strength at 3, 28 and 56 days. Compared with PVA fibers, BF has better effect on compressive strength and flexural strength, but it is not as effective as expected on splitting tensile strength.
(2) As a whole, the 28 days specimens show the greatest decrease in strength with the increase of W/B. When W/B changes from 0.28 to 0.40, the 28 days compressive strength, splitting tensile strength and flexural strength decrease by 17%, 13% and 27%, respectively. In terms of compressive strength and splitting tensile strength, the effect of W/B on BF-ECC is less than that on PVA-ECC.
(3) The pozzolanic effect of FA decreases the strength of BF-ECC at 3 days but has a significant positive effect on the splitting tensile strength and flexural strength at 28 and 56 days. FA with a content of 50–60% mostly improves the strength of the BF-ECC.
(4) Along with the increase of the fiber volume fraction, W/B and FA content, the static modulus of BF-ECC almost all show a downward trend.
(5) The SEM images indicate that the BF are randomly distributed in three dimensions and combine well with the matrix. The presence of BF can effectively prevent the growth of cracks, thus improving the strength of ECC.

BF-ECC has the potential to be used to reduce the risk of fire in buildings and Bridges. Based on this research, a reference can be provided for the application of BF-ECC.

## Figures and Tables

**Table 1:** Chemical and physical properties of cement and fly ash (FA).

Chemical Composition (%)	Cement	FA	Physical Properties	Cement	FA
SiO_2_	21.08	66.43	Water required (%)	/	93.2
Al_2_O_3_	5.48	19.05	Specific gravity	3.12	2.32
Fe_2_O_3_	3.93	4.42	Retained on 45 μm (%)	4.2	4.3
CaO	62.27	3.08	-	-	-
MgO	1.78	1.25	-	-	-
SO_3_	2.65	0.28	-	-	-
Na_2_O	0.52	-	-	-	-

**Table 2:** Properties of basalt fiber (BF).

Length (mm)	Diameter (μm)	Young’s Modulus (GPa)	Elongation (%)	Tensile Strength (MPa)	Density (g/cm^3^)
12	16	100	4	2800	2.68

**Table 3:** Mix proportion of Engineered cementitious composite (ECC) mixtures (kg/m^3^).

Mixture ID	BF	Cement	FA	Sand	Water	HRWR
1-1	0	421	632	684	295	6.3
1-2	13.0	421	632	684	295	8.4
1-3	26.0	421	632	684	295	10.5
1-4	39.0	421	632	684	295	12.6
1-5	52.0	421	632	684	295	14.7
2-1	52.0	421	632	684	337	12.6
2-2	52.0	421	632	684	379	10.5
2-3	52.0	421	632	684	421	8.4
3-1	52.0	737	316	684	295	14.7
3-2	52.0	632	421	684	295	14.7
3-3	52.0	526	526	684	295	14.7
3-4	52.0	316	737	684	295	14.7
